# Using an experiential learning model to teach clinical reasoning theory and cognitive bias: an evaluation of a first-year medical student curriculum

**DOI:** 10.1080/10872981.2022.2153782

**Published:** 2022-12-01

**Authors:** Justin J. Choi, Jeanie Gribben, Myriam Lin, Erika L. Abramson, Juliet Aizer

**Affiliations:** a Division of General Internal Medicine, Weill Cornell Medicine, New York, NY, USA; b Department of Medicine, Weill Cornell Medicine, New York, NY, USA; c Division of Rheumatology, Hospital for Special Surgery, New York, NY, USA; d Department of Pediatrics, Weill Cornell Medicine, New York, NY, USA; e Department of Population Health Sciences, Weill Cornell Medicine, New York, NY, USA

**Keywords:** Clinical reasoning, cognitive bias, experiential learning, dual process theory, curriculum

## Abstract

**Background:**

Most medical students entering clerkships have limited understanding of clinical reasoning concepts. The value of teaching theories of clinical reasoning and cognitive biases to first-year medical students is unknown. This study aimed to evaluate the value of explicitly teaching clinical reasoning theory and cognitive bias to first-year medical students.

**Methods:**

Using Kolb’s experiential learning model, we introduced dual process theory, script theory, and cognitive biases in teaching clinical reasoning to first-year medical students at an academic medical center in New York City between January and June 2020. Due to the COVID-19 pandemic, instruction was transitioned to a distance learning format in March 2020. The curriculum included a series of written clinical reasoning examinations with facilitated small group discussions. Written self-assessments prompted each student to reflect on the experience, draw conclusions about their clinical reasoning, and plan for future encounters involving clinical reasoning. We evaluated the value of the curriculum using mixed-methods to analyze faculty assessments, student self-assessment questionnaires, and an end-of-curriculum anonymous questionnaire eliciting student feedback.

**Results:**

Among 318 total examinations of 106 students, 254 (80%) had a complete problem representation, while 199 (63%) of problem representations were considered concise. The most common cognitive biases described by students in their clinical reasoning were anchoring bias, availability bias, and premature closure. Four major themes emerged as valuable outcomes of the CREs as identified by students: (1) synthesis of medical knowledge; (2) enhanced ability to generate differential diagnoses; (3) development of self-efficacy related to clinical reasoning; (4) raised awareness of personal cognitive biases.

**Conclusions:**

We found that explicitly teaching clinical reasoning theory and cognitive biases using an experiential learning model provides first-year medical students with valuable opportunities for developing knowledge, skills, and self-efficacy related to clinical reasoning.

## Introduction

Clinical reasoning, which can be defined as the cognitive processes clinicians employ to diagnose and treat patients, is an integral component of professional competence among physicians and trainees[1]. Cognitive errors, especially those associated with common cognitive biases in medicine (e.g., anchoring bias, availability bias, premature closure), contribute to a majority of diagnostic errors in clinical practice [2–8]. A national survey of medicine clerkship directors in the USA reported that the majority of medical students entering medicine clerkships have a fair or poor understanding of clinical reasoning concepts[9]. Most respondents reported that a structured curriculum in clinical reasoning should be taught across the medical education continuum, including the pre-clerkship years. Furthermore, to address the harms of diagnostic errors in clinical practice, the National Academies of Sciences has called for the creation of explicit, theory-informed clinical reasoning curricula in undergraduate and graduate medical education [10,11].

However, it remains uncertain whether clinical reasoning theories and cognitive biases can be effectively taught to pre-clerkship medical students given their relatively limited clinical knowledge and lack of clinical experience [12–15]. Medical education based on clinical reasoning theories and cognitive biases has mostly targeted learners with clinical experience [13,14,16–24]. Teaching of clinical reasoning theories has focused on dual process theory and script theory, and some studies have demonstrated improved diagnostic performance among senior medical students and residents [25–31]. Dual process theory is a model of problem solving that posits two systems of thinking: an intuitive and automatic processing that uses heuristics and pattern recognition (System 1), and a more analytic and deliberate processing (System 2)[32,33]. Script theory describes the reorganization of encapsulated knowledge of diseases, conditions, or syndromes into illness scripts, which are cognitive models of disease states that include the predisposing conditions, pathophysiological insults, and clinical consequences [34–37]. In diagnostic clinical reasoning, these theories are manifested in teaching learners how to: (i) formulate a *problem representation* (i.e., a succinct, abstract summary of the most defining features of the case); (ii) use *semantic qualifiers* (i.e., opposing descriptors of clinical features that help distinguish between diagnostic considerations); and (iii) search and select for *illness scripts* that most closely match their representation of a case[38].

The implementation of a clinical reasoning curriculum teaching dual process theory, script theory, and cognitive biases to first-year medical students has not been described in the literature. Here we describe our design and implementation of a clinical reasoning curriculum in the first year of medical school that explicitly introduces these theories and common cognitive biases that affect diagnostic reasoning.

We used Kolb’s model of experiential learning to guide the design and evaluation of the clinical reasoning curriculum. According to Kolb, the creation of knowledge (i.e., learning) results from a process of obtaining and transforming experiences[39]. In Kolb’s model, learning takes place in a four-stage cycle: (1) concrete experience, whereby learners engage in an experience; (2) reflective observation, in which learners observe and reflect on the experience; (3) abstract conceptualization, whereby learners integrate their observations with prior knowledge to form conclusions; and (4) active experimentation, in which learners test their new conceptual understanding. We delivered *concrete experiences* with a series of three written cases with both small group and individual *reflections* after each case. We built in opportunities for the students to *conceptualize* the clinical reasoning process, learn from their experience, plan and *experiment* with new strategies and behaviors to apply to future cases in the curriculum.

The purpose of this study was to evaluate the value of this theory-informed clinical reasoning curriculum for first-year medical students’ development of clinical reasoning and recognition of cognitive biases. Herein, we also describe the design and implementation of our experiential curriculum for teaching clinical reasoning concepts, theories, and cognitive biases to first-year medical students.

## Methods

### Setting and context of the curriculum

In 2014, Weill Cornell Medicine (New York, New York) restructured its medical school curriculum by dividing undergraduate medical education into three phases: (1) a foundational curriculum in the first year and a half, integrating basic sciences with clinical medicine delivered in organ system units; (2) clinical clerkships starting in the second half of the second year; and (3) a post-clerkship curriculum including clinical electives, sub-internships, courses in advanced clinical ethics and healthcare systems, and time dedicated to scholarly work in an area of concentration. In the first-year foundational curriculum, students gain some clinical experience with real patients in the LEAP (Longitudinal Educational Experience Advancing Patient Partnerships) program, which assigns first-year medical students to patients with whom they meet monthly in the context of a medical office visit, hospitalization, home visit, or phone call. In addition, students have clinical preceptorships in which they elicit medical histories and practice elements of the physical exam, and the organ system units include patient panel presentations and case-based small group sessions focused on pathophysiology within a single organ system.

### Description of the curriculum

#### Didactic lectures introducing clinical reasoning concepts

The curriculum (Table 1) began with two one-hour didactic lectures on diagnostic clinical reasoning, including concepts of dual process theory, problem representation, illness scripts, semantic qualifiers, and cognitive biases: anchoring bias, availability bias, base-rate neglect, confirmation bias, diagnostic momentum, implicit bias, representativeness bias, and premature closure. Lectures included an exercise that prompted students to generate illness scripts. Interactive case discussions helped students recognize cognitive biases in their clinical reasoning and apply specific clinical reasoning strategies, including diagnostic verification, worst-case scenario medicine, asking ‘why?’, and diagnostic frameworks.

**Table 1.** Curriculum timeline.

Year 2020	January	February			March			April			May			June	
Foundational Curriculum	Cardiology Unit	Pulmonary Unit		Gastro-intestinal Unit			Kidney Unit		Hematology/ Oncology Unit			Endocrinology Unit		Repro-duction Unit	
Clinical Reasoning Curriculum	Intro Lectures	Practice Assign-ment	CRE 1							CRE 2			CRE 3		

Abbreviation: CRE, Clinical Reasoning Examination.

These lectures were delivered in the third week of January 2020, at the start of the second semester of the students’ first year of medical school. The lecturer (JC) has received faculty development training in clinical reasoning education and at least three years of experience co-leading clinical reasoning workshops and courses for medical students, residents, and junior faculty at Weill Cornell. Subsequently, a clinician-educator with evidence-based medicine curriculum training and a medical librarian led a session on how to formulate background and foreground questions of a clinical problem.

A written case-based assignment was delivered after these introductory lectures for students as an exercise for formulating a problem representation, generating illness scripts, justifying their diagnostic reasoning, identifying background and foreground questions prompted by the case, and describing search strategies for the best evidence to address these questions.

#### Clinical reasoning examinations

A series of three full-day clinical reasoning examinations (CREs) were held bimonthly over the semester (February, April, June 2020). For each CRE, students were given Part 1 of a written clinical case. Part 1 included a History & Physical Examination (H&P) note that provided case information from the chief concern through diagnostic testing results as one would read in a standard hospital admission note (omitting the assessment and plan sections). This ‘whole-case’ approach allowed for standardization of case information available to each student, avoiding reliance on students to elicit the history given their limited experience eliciting histories from patients. Cases were more complex than those used in other case-based learning small groups and integrated content from the organ systems the students had learned about to date (the first CRE involved the heart and the lungs; the second added gastrointestinal and renal processes, and the third added hematology/oncology and endocrine conditions). Cases were peer-reviewed by the organ system unit leaders.

Students were allowed two hours to individually review Part 1 of the case (without access to resources) and submit written responses to Part 1 questions adapted from the IDEA Assessment Tool [40], which asked the following: (1) Write a problem list; (2) Write a problem representation; (3) Provide illness scripts for each of the three most likely causes of this patient’s presentation; (4) Provide your explanation for your leading hypothesis; (5) Describe your reservations about your leading hypothesis; (6) Address alternative hypotheses – what features of the case are consistent and/or not consistent with each hypothesis?; (7) Describe additional information needed or further diagnostic workup; and (8) Provide a background question that would help with your diagnostic reasoning in this case, and an appropriate resource to look for the answer. Examples of a problem representation and illness script generated as part of the CRE are provided in the Supplementary Materials.

After submitting their written responses, students received Part 2 of the case. Part 2 provided additional case information with additional diagnostic investigations that were pursued. Students were given three hours to review this additional case information, access resources, and collaborate with classmates (if desired). Then, students submitted written responses to the following prompts: (1) Present a framework or systematic approach for the main clinical problem in the case; (2) Provide an assessment that includes an updated problem representation, your leading diagnosis, and rationale supporting your leading diagnosis and refuting alternatives; and (3) Formulate a foreground question prompted by this case and describe an appropriate resource to answer this question. All written responses to the Part 1 and Part 2 questions were submitted electronically.

Each CRE concluded with a 90-minute small group session with approximately 10–12 students and a faculty facilitator to review and discuss Part 1 and Part 2 of the case. Faculty facilitators were recruited from the Department of Medicine and included academic hospitalists, general internists, and medicine subspecialists. The small group sessions for the first CRE were held in-person. Due to the COVID-19 pandemic, the small group sessions for the second CRE were held virtually, and a virtual large-group format was used for the third CRE.

During the weeks after the CRE, students had an opportunity to review sample answers to the Part 1 and Part 2 prompts for a time-limited period and were not allowed to copy or share the sample answers. This policy was enforced to allow delivering the same CRE case materials in subsequent years of the new curriculum.

#### Student self-assessment questionnaires

After each CRE, students viewed a sample answer and were given two weeks to complete a self-assessment questionnaire prompting them to: (1) Identify any missing key components in their Part 1 problem representation (patient’s age and gender, relevant medical history, relevant symptoms, the temporal onset of the illness, relevant signs of physical examination, relevant data); (2) Identify any semantic qualifiers that they used in the problem representation; (3) Identify if their problem representation had excess verbiage; (4) Identify knowledge gaps that limited their diagnostic reasoning; (5) Describe how cognitive biases might have affected their diagnostic reasoning; (6) Describe strategies or resources used during the CRE that were helpful; (7) Describe strategies that might improve their diagnostic reasoning in the future. The third and final CRE self-assessment questionnaire did not repeat questions on the problem representation and semantic qualifiers to focus the students on the higher-level questions regarding cognitive biases, learning strategies, and how their approach to clinical reasoning changed over the semester.

#### Faculty assessment questionnaires

Two faculty evaluators (JC and JA) completed an assessment of each student’s written assignment for whether the problem representation was: (1) complete; (2) concise (i.e., did not have excess verbiage); and (3) included semantic qualifiers (see Supplemental Materials for an example of a complete and concise problem representation with use of semantic qualifiers that was provided to students in the sample answer sheet). The faculty assessment questionnaire also evaluated the student self-assessment questionnaire to: (1) determine whether the student accurately recognized if their problem representation had missing components and/or excess verbiage; (2) determine whether student responses demonstrated any confusion in their identification of semantic qualifiers; (3) determine whether they correctly identified and/or named a cognitive bias; and (4) provide feedback and comments on their self-assessment in general. The faculty evaluators both independently reviewed 20% of students’ written assignments and self-assessment questionnaires, then compared their evaluations to reach a consensus on how to adjudicate and/or provide feedback on elements of students’ written responses. They then divided the remaining written assignments for review using the faculty assessment questionnaire. The faculty assessment questionnaire responses were delivered to students for formative feedback. The CREs were graded as Pass/Fail based on a global assessment by the faculty evaluators for satisfactory completion of Part 1, Part 2, and the self-assessment questionnaire.

#### Anonymous questionnaire

An anonymous questionnaire was administered at the conclusion of the curriculum (June 2020) to gather student feedback. Students were asked to rate the extent to which they felt the CREs were a valuable learning experience (0: not at all; 1: to a small extent; 2: to a moderate extent; 3: to a large extent). They were also asked to provide free-text responses to the following open-ended questions: (1) What, if anything, did you find valuable about the CREs?; (2) What was it like to learn about cognitive biases and clinical reasoning strategies this semester? (3) Do you think cognitive biases and/or clinical reasoning strategies should be taught earlier or later in the curriculum? If so, what should be taught when, and why?; (4) What issues, if any, did you encounter when trying to discuss any of the CRE cases with other students?; (5) In what ways, if any, did you have to adjust your strategies for Part 2 as a result of the distance format?; (6) The case discussions with faculty took the form of in-person small group sessions for the first CRE, virtual small group sessions for the second, and a virtual large group session for the third. Please comment on your experience in these sessions, including any effect of the format on your experience.; and (7) Please share any suggestions for how to improve the CREs or any other aspects of the clinical reasoning curriculum.

### Evaluation of the curriculum and impact

#### Participants and protocol

All first-year medical students (N = 106) at Weill Cornell Medicine in the 2019–2020 academic year were included in this study. The study was determined to be exempt from full review by the Weill Cornell Medicine Institutional Review Board (20–02021546). Given that this study was considered educational research and exempt from federal regulations governing research, notification to students of study information and informed consent were not required. The research team consisted of two faculty leaders of the curriculum (JC, JA), two co-investigators not involved in the curriculum (JG, EA), and a research assistant (ML).

#### Evaluation measures

*Clinical reasoning performance* To assess the medical students’ clinical reasoning performance, we evaluated data from the students’ written CRE submissions, self-assessment questionnaires, and faculty assessment questionnaires to determine: (1) whether the problem representation was considered complete; (2) whether the problem representation was considered concise; (3) whether semantic qualifiers were used in the problem representation; and (4) whether the student self-assessment demonstrated any confusion on semantic qualifiers.

*Cognitive biases* To assess the medical students’ ability to identify and understand cognitive biases in their clinical reasoning, we evaluated the student self-assessment questionnaires to determine whether students identified one or more cognitive biases by name and/or described one or more cognitive biases in their response. We also assessed for any occurrences of students’ misunderstanding cognitive biases in their self-assessment questionnaires (e.g., identifying a bias by name, but then describing it incorrectly in their response; describing a phenomenon that is not a cognitive bias).

*Clinical reasoning and learning strategies* To assess the medical students’ reflections on strategies in their learning or clinical reasoning during the CRE experience and plans for applying new strategies (i.e., experimenting), we evaluated the student self-assessment questionnaire to determine: (1) whether clinical reasoning/learning strategies and/or resources were used; and (2) whether they planned to use a particular strategy to improve their clinical reasoning in the future.

*Students’ perspectives on the curriculum* To assess students’ perspectives on the curriculum, we evaluated students’ responses to the anonymous questionnaire delivered at the end of the curriculum to determine: (1) the extent to which students felt the CREs were a valuable learning experience, (2) what students found valuable about the CREs; (3) how students experienced learning cognitive biases and clinical reasoning concepts over the semester; (4) whether students thought this was the optimal timing in the medical school curriculum for teaching cognitive biases or clinical reasoning concepts; (5) what issues students encountered in trying to discuss the cases with classmates; (6) what adjustments students made as a result of the virtual format in the second and third CREs; (7) whether students preferred a particular format for case discussions with faculty; and (8) what suggestions students had for improvement of the curriculum.

#### Data collection

Study data were collected and managed using REDCap electronic data capture tools hosted at Weill Cornell Medicine [41,42]. Student self-assessment questionnaires were administered electronically immediately following each CRE. Faculty assessment questionnaires were administered electronically approximately two weeks after each CRE when students’ written assignments and self-assessments were organized and compiled by a curriculum coordinator. All data were collected as part of the standard curriculum and de-identified prior to data analysis – a research assistant removed all students’ names and assigned unique identification numbers at random to each student (from 1 to 106).

#### Data analysis

Descriptive statistics were calculated for quantitative data relating to the evaluation measures of medical students’ clinical reasoning, cognitive biases, clinical reasoning/learning strategies, and the impact of the curriculum. We did not use any statistical tests of association to make comparisons between CREs because clinical reasoning performance is highly case- and context-specific, meaning that any statistically significant differences in performance between CREs would likely be attributed to differences in cases and/or context of the CREs [12,43–45].

We used manifest content analysis, defined as describing what is occurring on the surface without the need to discern intent or identify deeper meaning (‘staying close to the text’) [46,47], for student responses to open-ended questions on cognitive biases, strategies, or resources that were used during the CRE, proposed strategies or resources to use in future cases, and student reflections on how their approach to clinical reasoning changed over the semester.

Content analysis was performed in three main phases: familiarization with the data, categorization of the data (assigning codes to relevant data segments and organizing codes into categories and subcategories), and reporting the study findings[48]. A group of four investigators (JC, JG, EA, JA) initially met to familiarize everyone with the data. Categorization of the data was initially conducted in pairs (JC/JG and EA/JA), then as a group to review and discuss disagreements that would be settled by consensus. The leaders of the curriculum (JC and JA) were paired with investigators who were not involved in designing or delivering the curriculum (JG and EA). Several rounds of this approach were performed until the group felt confident in the consistency of their coding, after which the remainder of the sample was distributed equally to each pair for the final categorization of the data.

## Results

### Clinical reasoning performance

Among a total of 318 CREs that were completed by 106 students, 254 (80%) had a complete problem representation and 199 (63%) of problem representations were considered concise. Semantic qualifiers were included in 195 of 212 (92%) problem representations analyzed in the first two CREs. Only 1 of every 7 students’ self-assessment responses demonstrated confusion about semantic qualifiers.

### Cognitive biases

Among all student self-assessments of their clinical reasoning during the CREs, 261 of 318 (82%) showed that students were able to identify or describe a cognitive bias (Supplemental Table 1). The most common cognitive biases across all 3 CREs were anchoring bias, availability bias, and premature closure. Anchoring bias is the tendency to ‘lock onto’ salient features in the patient’s initial presentation too early in the diagnostic process and failing to adjust this initial impression as new information becomes available[49]. Students were able to identify key features of the case that immediately led them to a specific diagnostic hypothesis:

I always automatically associate unintentional weight loss with malignancy, but this kind of stereotyping/anchoring might limit my differential diagnosis. (P101)

The heavy alcohol consumption caught my attention, so I was immediately suspicious of alcohol-induced liver disease while reading the case. (P27)

Availability bias is the tendency to judge things as being more likely if they readily come to mind (e.g., a recent experience with a disease)[49]. Students mostly identified their concurrent organ system units and lecture content as sources of availability bias:

We had just finished the renal unit, so I spent quite a bit of the time in Part 1 thinking about all the possible renal causes … even though the patient didn’t have any of the classic renal signs like hematuria or proteinuria. (P21)

In lecture it was mentioned that carcinoid could be associated with tricuspid regurgitation. When I saw tricuspid regurgitation, I immediately thought of carcinoid. (P74)

Premature closure is the tendency to stop considering other possibilities after reaching a diagnosis and is the single most common cognitive bias in diagnostic errors[2]. Because it is often the ‘final common pathway’ for cognitive biases leading to diagnostic errors, we adjudicated premature closure in descriptions that did not have another cognitive bias described ‘upstream’ to premature closure (e.g., anchoring bias that led to premature closure):

The patient had all the signs of liver failure and acute tubular necrosis, so I ended my reasoning there. This caused me to miss the inciting incident, which was spontaneous bacterial peritonitis. (P59)

Representativeness bias and confirmation bias were also identified and/or described by students. Representativeness bias is the tendency to be drawn toward prototypical manifestations of disease[49].

The patient’s symptom of angina radiating to the left shoulder, along with labs showing troponin elevation and ECG T inversion, which are all defining characteristics of myocardial infarction, convinced me wrongly that the patient had an MI. (P29)

Confirmation bias is the tendency to look for confirming evidence to support a diagnosis rather than look for disconfirming evidence to refute it[49].

When I looked at the EKG, I expected to find an ST elevation, so that’s what I assumed that I saw (even though it was an ST depression).(P56)

We identified several students describing a ‘test-taking’ bias in each CRE, which we defined as any influence on reasoning caused by personal reactions, behaviors, and strategies when taking the test:

I was primarily focused on diagnoses within Endocrine, Repro, and Heme/Onc, as these were the units that had not been previously covered on CREs. While this was an effective test-taking strategy, this same reasoning could obviously not be applied to real patients. (P57)

In general, students infrequently identified cognitive biases by name. We found only four occurrences across all CREs in which the student self-assessment demonstrated confusion in their attempt to identify and/or describe a cognitive bias. Approximately 20% of students did not identify or describe any cognitive bias affecting their reasoning in each CRE.

### Clinical reasoning and learning strategies

Students described using illness scripts and diagnostic frameworks more frequently than other clinical reasoning strategies such as diagnostic verification, worst-case scenario medicine, and asking ‘why?’ strategies (Supplemental Table 2). During each CRE, students frequently collaborated with peers and reviewed other resources (e.g., lecture notes, online resources). In planning for future CREs, students commonly mentioned their intentions to broaden their diagnostic thinking.

Methods for reading the case materials, including highlighting, underlining, and annotating were mentioned in student self-assessments, but we did not consider any to be strategies for clinical reasoning. Strategies that were too vague to categorize, such as focusing or systematically reviewing case material without specifying how, and generally avoiding cognitive biases, were also not considered descriptions of strategies for clinical reasoning in our analysis.

### Students’ perspectives on the curriculum

Seventy-seven (73%) students responded to the anonymous questionnaire that assessed their perspectives on the curriculum. The majority of respondents rated the extent to which the learning experience in CREs was valuable as great (71%, 55/77) and moderate (26%, 20/77). Only 2 of 77 respondents found the learning experience to have small or no value.

Four major themes emerged as valuable outcomes of the CREs as identified by students: (1) synthesis of medical knowledge; (2) enhanced ability to generate differential diagnoses; (3) development of self-efficacy related to clinical reasoning; (4) raised awareness of personal cognitive biases. These same themes were identified in student responses to the question on the final CRE self-assessment on how their approach to clinical reasoning changed over the semester.

#### Synthesis of medical knowledge

Students found that the CRE curriculum offered opportunities to integrate medical knowledge from multiple organ system units and apply prior knowledge acquired in earlier units of the overall first-year curriculum:

Since we learn clinical knowledge in an organ system-based way in [the first-year curriculum], it sometimes can be difficult to integrate that information. CREs have been a great opportunity for me to try and combine knowledge from different organ systems and think critically about a complex clinical case, in a way that we don’t have a lot of other opportunities to do in other parts of the curriculum. (P37)

#### Enhanced ability to generate differential diagnoses

Many students found that the CREs made explicit the generation of a differential diagnosis, and regarded this as valuable practice for clinical problem solving:

I find that they really help me hone in on how to form a directed differential diagnosis. Learning things [by organ system] is great for an initial pass, however, it can inhibit you from looking at things holistically when faced with a real patient. I like that this exercise forces us to analyze every system at the same time to formulate a [differential] diagnosis. (P32)

#### Development of self-efficacy related to clinical reasoning

Self-efficacy can be defined as the belief that one can successfully perform a specific task. It is one of the strongest motivators for learning, which could be targeted as a strategy for improving diagnostic clinical reasoning[50]. Students found that the CREs helped them better understand and apply clinical reasoning skills through practice, and felt more prepared for clinical reasoning in the real clinical environment:

Good practice cases for incorporating multiple units (since we don’t have any other cumulative exams), helping understand [the] diagnostic approach, and feeling more ready [sic] to not only diagnose, but present cases. (P18)

#### Raised awareness of personal cognitive biases

Students believed that their awareness of personal cognitive biases in clinical reasoning was enhanced by the CREs. Students expressed appreciation for learning how to identify cognitive biases, how to potentially mitigate their cognitive biases, and how to improve their diagnostic reasoning by exposing their cognitive biases.

Taking the time to practice diagnosing […] and how to avoid our biases while diagnosing was a very valuable experience. (P56)

In addition to how the CREs impacted their clinical reasoning performance, students also found value in the format and methodology of the CRE curriculum, including: (1) opportunities for application and practice with clinical reasoning; (2) learning from cases with complexity and high-fidelity; (3) open-ended questions and discussion format; (4) learning from peers; and (5) learning from clinicians.

#### Students’ experience learning cognitive biases and clinical reasoning

Most students who responded to the open-ended question of what it was like to learn about cognitive biases and clinical reasoning concepts over the semester either believed they became better equipped to recognize and address their own biases (36%, 28/77) or believed they had improvement in their diagnostic skills or clinical reasoning (30%, 23/77). Students found it helpful to raise awareness of their own cognitive biases, particularly in a low-stakes environment in which feedback and errors were welcome for self-improvement:

I found it helpful to identify cognitive biases I personally had that I was previously unaware of. It certainly helped me to identify these now, especially since the stakes are low, because I have been more cognizant of addressing these as the semester progressed. The CRE’s were a fun, low-stress way to practice clinical reasoning. I was often more happy when I was wrong in fact, because we got feedback immediately afterwards and I was able to adjust my reasoning for the next time rather than practicing in real time with real patients. (P02)

Some students realized they were prone to premature closure (12%, 9/77). They also believed they became less susceptible to premature closure through the CRE experience:

It was definitely a revelation. I didn’t realize how easy it was, even while coming up with multiple hypotheses, to get pigeon holed into one way of thinking. (P56)

It was useful to train myself to keep an open mind and consider various differential diagnoses rather than becoming hooked on one too early. (P22)

#### Timing in the medical school curriculum

The majority of respondents (70%, 54/77) felt that cognitive biases and/or clinical reasoning strategies were being taught at the right time in the medical school curriculum, in the second semester of first year, and 14% (11/77) believed these topics should be taught earlier in the curriculum. Only one student believed these topics should be taught later in the curriculum; 11 did not respond to the question regarding curricular timing.

#### Issues encountered in peer case discussions

Students recognized biases in peer discussions, demonstrating the ability to not only critique their individual strategies, but also their collaborative approaches. Fourteen respondents (18%) described how discussing cases with peers either enabled them to identify potential cognitive biases in peers or affected their own clinical reasoning. For example, students identified cognitive biases that prompted them to reflect on an ideal state for the diagnostic process:

I think that some students cling tightly to their views and hypotheses in a black and white, right or wrong way, when in reality these differentials need to be fluid. (P15)

Other students identified potential ‘group biases’ such as *group polarization* (when groups make more extreme judgments and decisions than the initial position of its individual members), *groupthink* (when group harmony and cohesion leads to premature consensus and may inhibit the expression of individual opinion), and *social loafing* (when individuals feel ‘lost in the crowd’ and may have a reduced level effort in group situations) [51]:

One person comes up with a crazy, zebra idea, which leads to multiple people latching on, then the crazy, zebra idea becomes main idea of the group. (P51)

Groupthink was pretty prevalent. I noticed that a lot of times, it was easy to just put aside the diagnoses that stood out and were not included in all of our illness scripts during the conversations. (P68)

When groups were large enough, it was easy to get a bit lost in so many ideas and discussions—particularly if I had a knowledge gap and was not able to follow the thought process. (P70)

Thirteen respondents (17%) identified at least one negative aspect of the curriculum. Most negative impressions related to the introductory didactic lectures. Students found that the lectures on clinical reasoning and cognitive biases were ineffective compared to the practical experience of the CREs:

I think practical exercises, like the CREs, are better at teaching cognitive bias and clinical reasoning than lectures. Even if I understand something intellectually, I don’t think I can effectively use the concepts without practice. (P66)

#### Adjustments due to a virtual format and preferred format

In response to any adjustments made in their clinical reasoning or learning strategies resulting from the virtual format for Part 2 (peer discussions) in the second and third CREs, the majority of respondents (60%; 46/77) indicated that they used a virtual platform to discuss cases with peers (e.g., Zoom, FaceTime). Regarding the various case discussion formats that concluded each CRE, 40% (31/77) of respondents felt that either the in-person small groups or virtual small groups were the best for case discussions; 40% (31/77) regarded virtual small groups as ‘the best’ (and favored over the in-person small group format); 31% (24/77) regarded the in-person small groups as ‘the best’ (and favored over the virtual small group format). Eleven students (14%) felt that all three formats were equally ‘the best.’ However, 38% (29/77) did not like the virtual large group, whereas only nine students did not like the virtual small groups and only three students did not like the in-person small groups.

#### Suggestions for improvement

Free text responses to a prompt asking for suggestions for improving the curriculum fell into the following categories: would not change anything (44%, 34/77); have more CREs (25%, 19/77); improve the group discussions (14%, 11/77); have shorter CRE days (12%, 9/77); improve the quality of the CRE case content (10%, 8/77); eliminate grading (8%, 6/77); reinforce/expand on clinical reasoning in the organ units (4%, 3/77); provide better preparation students for the first CRE (5%, 4/77); and improve the feedback for students (4%, 3/77).

## Discussion

We used Kolb’s model of experiential learning to develop and implement a theory-informed clinical reasoning curriculum for first-year medical students. Students were introduced to clinical reasoning theory and cognitive biases, provided with experiences for clinical reasoning, and were prompted for reflection, conceptualization, and iterative experimentation with clinical reasoning concepts, strategies, and cognitive biases.

All students progressed through Kolb’s first stage of learning with new experiences in diagnostic clinical reasoning in the form of a series of didactics followed by a CRE. Students immersed themselves in the content both individually and collectively with peers and in small groups with faculty. Most students demonstrated the ability to formulate a complete problem representation, include semantic qualifiers, and identify or describe cognitive biases in their clinical reasoning. Students indicated they felt the content was appropriately placed in the first-year curriculum and could be flexibly transitioned to a virtual experience if necessary.

In Kolb’s second stage of experiential learning, students reflected on the CRE through self-assessments. Students most frequently described anchoring bias, availability bias, and premature closure in their diagnostic clinical reasoning process. Students reported using illness scripts and diagnostic frameworks more commonly than other clinical reasoning strategies (such as meta-cognitive strategies of asking ‘why?’ or practicing ‘worst-case scenario medicine’). Students also found collaboration with peers and use of online resources or lecture materials to be helpful.

In Kolb’s third stage of experiential learning, students conceptualized the diagnostic clinical reasoning process and were able to describe strategies for improving their approach to clinical reasoning. In addition, students thought that this curriculum promoted their synthesis of medical knowledge, enhanced their ability to generate differential diagnoses, developed their self-efficacy related to clinical reasoning, and raised their awareness of personal cognitive biases.

In Kolb’s fourth stage of experiential learning, students had opportunities to experiment with new strategies and behaviors in subsequent CREs. Students most frequently mentioned their intentions to ‘broaden their diagnostic thinking’ in subsequent encounters. Future investigations should explore how medical students attempt to broaden their diagnostic thinking and perform comparative studies of different techniques or strategies that lead to improved clinical reasoning performance.

To our knowledge, our curriculum is the first detailed description of how clinical reasoning theories and understanding of cognitive biases can be introduced to first-year medical students. A systematic review of pre-clinical education programs teaching illness scripts found two studies that were directed at first-year medical students[52]. Hennrikus et al. tasked students to write illness scripts for the diseases they were learning during problem-based learning and lectures and to reflect upon them[53]. Jackson et al. developed a simulated clinic activity for first-year medical students that aimed to have students learn illness scripts of various viral diseases[54]. However, neither introduced the underlying clinical reasoning theories, cognitive biases, and strategies for avoiding these biases. We found that explicit teaching of clinical reasoning theories and cognitive biases paired with experiential learning cycles involving challenging written cases led to high performance in essential elements of diagnostic clinical reasoning: 80% demonstrated complete problem representations and 82% identified or described cognitive biases. The lowest scoring element was the ability to write a concise problem representation (63%), which should be a target for future study and intervention to improve first-year medical students’ diagnostic clinical reasoning.

Evidence demonstrating the effectiveness of educational interventions focused on raising awareness of reasoning and cognitive biases has been limited[55]. However, experimental studies in psychological sciences have shown that debiasing-training interventions can have long-lasting effects on improving decision making, including among graduate students [56,57]. Cognitive debiasing occurs through a succession of stages from precontemplation, to awareness and the ability to detect bias, to the decision to change, then initiation of strategies to accomplish and maintain the change[58]. Lack of awareness is one factor that may explain the difficulties in mitigating cognitive biases. Raising awareness of cognitive biases in the first year of medical school has several potential benefits: it extends the overall time that students can develop their knowledge, skills, and attitudes on cognitive biases longitudinally; it provides dedicated time for students to focus on developing these skills and perspectives *before* the competing demands and cognitive load on clerkships; it provides the opportunity for students to see the relevance of cognitive biases in subsequent material and clinical encounters; and it allows students to practice with an experiential learning model that routinely involves reflection, conceptualization, and planning for the next encounter.

Our study also found that students valued developing their self-efficacy in clinical reasoning performance through opportunities to integrate their medical knowledge and generate differential diagnoses. In practicing clinical reasoning and recognizing their cognitive biases, students believed they became better equipped to address cognitive biases, improve their diagnostic clinical reasoning skills, and avoid premature closure. Self-efficacy is a critical, domain-specific phenomenon that may explain some of the variation in medical students’ sense of preparedness for professional activities during clerkships[59]. Introducing clinical reasoning concepts in the first year of medical school may be advantageous for building self-efficacy in clinical reasoning, easing their transition to clerkships.

Students also recognized, without prompting, the important role that group dynamics and potential ‘group biases’ may play in their learning and clinical reasoning performance. We found that students identified group biases such as group polarization, groupthink, and social loafing. These systematic biases in group decision-making have received some attention in patient safety, albeit with very few empirical studies[51]. Further research is needed to explore the role and influence of group biases in clinical reasoning and medical education.

Our study has some limitations. First, this is a curriculum intervention on a single group without a pre-post or concurrent group comparison. A pre-post comparison of clinical reasoning performance is not feasible for entering first-year medical students without any prior knowledge of clinical medicine or clinical reasoning. A concurrent comparison group was also not possible given that this was a single institution study. Second, self-assessment questionnaires were not anonymous, which might have influenced students to describe their cognitive biases or clinical reasoning strategies in a manner that might be viewed more favorably by the curriculum, however student responses on the anonymous questionnaire supported sentiments expressed on the self-assessment questionnaires. Third, there was a degree of subjectivity in the classification of cognitive biases described by students. We attempted to mitigate this limitation by meeting frequently, and reaching consensus through discussions, to ensure a shared understanding of cognitive bias definitions and approaches to adjudication. We also purposefully paired the curriculum leaders with ‘non-curriculum’ investigators to limit research bias among the teachers of clinical reasoning and cognitive biases.

## Conclusion

To our knowledge, this is the first study to describe and evaluate a clinical reasoning curriculum that introduces dual process theory, script theory, and cognitive biases in clinical reasoning to first-year medical students. We found that teaching clinical reasoning theory and cognitive biases using an experiential learning model provides first-year medical students with valuable opportunities for developing knowledge, skills, and self-efficacy related to clinical reasoning. Further research is needed to evaluate the effect of early clinical reasoning and cognitive bias training on medical students’ clinical reasoning performance during clerkships.

## Supplementary Material

Supplemental Material

## Data Availability

The data that support the findings of this study are available from the corresponding author, JC, upon reasonable request.
