# Supplementary material for: Using an experiential learning model to teach clinical reasoning theory and cognitive bias: an evaluation of a first-year medical student curriculum
Source: Med Educ Online. 2022 Dec 1;28(1):2153782. doi: 10.1080/10872981.2022.2153782 (PMC9718553; doi:10.1080/10872981.2022.2153782)
Supplement: Supplemental Material [file ZMEO_A_2153782_SM3349.docx]

**Example problem representation and illness script generated by a student.**

Problem representation:

*Mr. R is a 52-year-old man with hypertension, hyperlipidemia, and alcohol use disorder who presents with a 2-month onset of abdominal distension, abdominal pain, and jaundice, and for the last 2 days has developed altered mental status. On exam, he is hypotensive and diffusely jaundiced, somnolent with asterixis, has mild gynecomastia and spider angiomata on the anterior chest, moderate pitting edema bilaterally, and abdominal distention with a positive fluid wave and shifting dullness. Labs are significant for macrocytic anemia, thrombocytopenia, hyponatremia, acidosis, azotemia, increased creatinine, and abnormal liver functions particularly decreased albumin, elevated AST, ALT, alkaline phosphatase levels with a 2 AST: 1 ALT ratio, and increased bilirubin and INR.*

Illness scripts:

| **Illness Scripts:** | **Hypothesis #1**  *Alcoholic Hepatitis* | **Hypothesis #2**  *Hepatitis B virus infection* | **Hypothesis #3**  *Alcoholic cirrhosis* |
| --- | --- | --- | --- |
| **Epidemiology/**  **Predisposing Factors** | *Excessive alcohol intake* | *Risky sexual behavior*  *IV drug use* | *Excessive alcohol intake for many years* |
| **Time Course** | *Acute/Chronic* | *Acute/Chronic* | *Chronic* |
| **Pathophysiology** | *Inflammation of liver due to alcohol induced injury* | *Inflammation of liver due to Hep B virus* | *Steatosis 🡪 Hepatitis 🡪 Fibrosis 🡪 Cirrhosis 🡪 Portal hypertension* |
| **Clinical**  **Presentation**  **(Key symptoms, signs, diagnostics, and/or response to treatment)** | *Jaundice*  *Fatigue*  *Elevated liver function tests*  *2 AST: 1 ALT* | *Jaundice*  *Fatigue*  *Abnormal liver function tests*  *Hep B antigen positive (acute)*  *Hep B core antigen and antibody (chronic)* | *Cirrhosis 🡪 Jaundice, fatigue, abnormal liver function tests, edema, ascites, varices* |

**Examples of complete, concise problem representations with use of semantic qualifiers.**

The following are examples of complete problem representations with increasing levels of diagnostic reasoning skill and expertise demonstrated.

1. Example of a complete problem representation:

Mr. R is a 52-year-old man with hypertension, hyperlipidemia, obesity, and alcohol use disorder presenting with two weeks of abdominal pain and distension, generalized weakness, fatigue, and altered mental status. Exam is notable for relative hypotension, somnolence, jaundice, spider angiomata, palmar erythema, gynecomastia, abdominal distension with positive fluid wave and shifting dullness, peripheral edema, and asterixis. Labs are notable for low hemoglobin with high MCV, thrombocytopenia, elevated BUN and creatinine, transaminase elevation, hypoalbuminemia, and elevated INR.

2. Example incorporating semantic qualifiers (italics):

Mr. R is a 52-year-old man with hypertension, hyperlipidemia, obesity, and alcohol use disorder who presents with *subacute* onset of abdominal pain and distention, *generalized* weakness, fatigue, and altered mental status. Exam is notable for relative hypotension, somnolence, jaundice with scleral icterus, spider angiomata, palmar erythema, gynecomastia, *tense* ascites, *mild* and *diffuse* tenderness to palpation of abdomen, *moderate* and *bilateral* peripheral edema, and asterixis. Labs are notable for macrocytic anemia, thrombocytopenia, acute kidney injury, transaminase elevation in 2:1 AST > ALT ratio, hypoalbuminemia, and coagulopathy.

3. Example incorporating semantic qualifiers and combining related features:

Mr. R is a 52-year-old man with alcohol use disorder presenting with subacute abdominal pain and distension and altered mental status found to have signs of synthetic dysfunction of the liver and portal hypertension with tense ascites and hepatic encephalopathy as well as acute kidney injury.

**Supplementary Table 1. Cognitive biases self-identified and/described by students.**

|  | **N=106** |  |  |
| --- | --- | --- | --- |
|  | **CRE 1**  n (%) | **CRE 2**  n (%) | **CRE 3**  n (%) |
| None | 20 (19) | 19 (18) | 20 (19) |
| Anchoring Bias | 36 (34) | 51 (48) | 46 (43) |
| Identified by name | 7 (7) | 10 (9) | 9 (8) |
| Bias described without name | 31 (29) | 48 (45) | 39 (37) |
| Availability Bias | 14 (13) | 13 (12) | 22 (21) |
| Identified by name | 2 (2) | 6 (6) | 6 (6) |
| Bias described without name | 12 (11) | 12 (11) | 18 (17) |
| Representativeness Bias | 19 (18) | 4 (4) | 5 (5) |
| Identified by name | 2 (2) | 0 (0) | 1 (1) |
| Bias described without name | 18 | 4 (4) | 4 (4) |
| Confirmation Bias | 16 (15) | 15 (14) | 4 (4) |
| Identified by name | 3 (3) | 3 (3) | 1 (1) |
| Bias described without name | 14 (13) | 14 (13) | 4 (4) |
| Premature Closure | 15 (14) | 12 (11) | 12 (11) |
| Identified by name | 0 (0) | 0 (0) | 1 (1) |
| Bias described without name | 15 (14) | 12 (11) | 11 |
| “Test-Taking” Bias | 5 (5) | 3 (3) | 4 (4) |
| Identified by name | 0 (0) | 0 (0) | 0 (0) |
| Bias described without name | 5 (5) | 3 (3) | 4 (4) |

Other cognitive biases identified or described fewer than 5 times across all CREs were base-rate neglect (n=3), diagnostic momentum (n=2), and implicit bias (n=1).

**Supplemental Table 2. Clinical reasoning and learning strategies self-identified by students.**

|  | **N=106** | |  | |  | |
| --- | --- | --- | --- | --- | --- | --- |
|  | **CRE 1**  n (%) | | **CRE 2**  n (%) | | **CRE 3**  n (%) | |
|  | Used | Planned | Used | Planned | Used | Planned |
| Review other resources | 51 (48) | 7 (7) | 59 (57) | 2 (2) | 58 (55) | 8 (8) |
| Collaboration with peers | 44 (42) | 6 (6) | 39 (37) | 1 (1) | 44 (42) | 4 (4) |
| Illness scripts | 25 (24) | 19 (18) | 24 (23) | 15 (14) | 19 (18) | 34 (32) |
| Diagnostic frameworks | 15 (14) | 8 (8) | 8 (8) | 11 (10) | 16 (15) | 22 (21) |
| Forward reasoning | 13 (12) | 14 (13) | 10 (9) | 7 (7) | 7 (7) | 6 (6) |
| Probabilistic reasoning | 9 (8) | 5 (5) | 1 (1) | 3 (3) | 3 (3) | 6 (6) |
| Backward reasoning | 8 (8) | 6 (6) | 5 (5) | 4 (4) | 2 (2) | 6 (6) |
| Problem list development | 8 (8) | 1 (1) | 14 (13) | 3 (3) | 9 (8) | 3 (3) |
| Broaden diagnostic thinking | 7 (7) | 34 (32) | 6 (6) | 37 (35) | 2 (2) | 18 (17) |
| Attention to chronology | 2 (2) | 2 (2) | 4 (4) | 3 (3) | 2 (2) | 2 (2) |
| Causal reasoning | 2 (2) | 1 (1) | 3 (3) | 2 (2) | 0 (0) | 0 (0) |
| Worst-case scenario | 2 (2) | 1 (1) | 1 (1) | 1 (1) | 0 (0) | 0 (0) |
| Organ systems approach | 1 (1) | 4 (4) | 0 (0) | 0 (0) | 3 (3) | 1 (1) |
| Diagnostic verification | 1 (1) | 2 (2) | 4 (4) | 2 (2) | 3 (3) | 2 (2) |
| Asking “why?” | 1 (1) | 3 (3) | 1 (1) | 0 (0) | 1 (1) | 0 (0) |
